# Three-Chamber Images Are More Useful than Four-Chamber Images for the Volumetric Method of Degenerative Mitral Regurgitation

**DOI:** 10.3390/jcm15020807

**Published:** 2026-01-19

**Authors:** Ami Tateyama-Niwano, Haruka Sasaki, Hiroyuki Takaoka, Haruto Matsumoto, Kazuki Yoshida, Moe Matsumoto, Yoshitada Noguchi, Shuhei Aoki, Katsuya Suzuki, Satomi Yashima, Makiko Kinoshita, Noriko Suzuki-Eguchi, Kenji Kawasaki, Yoshio Kobayashi, Kazuyuki Matsushita

**Affiliations:** 1 Department of Laboratory Medicine, Chiba University Hospital, Chiba 260-8677, Japan; 2 Department of Cardiovascular Medicine, Chiba University Graduate School of Medicine, Chiba 260-8670, Japan

**Keywords:** degenerative mitral regurgitation, volumetric method, transthoracic echocardiography, three-dimensional transesophageal echocardiography

## Abstract

**Background/Objectives:** Effective regurgitant orifice area (EROA) is a critical parameter in assessing mitral regurgitation (MR) severity. The Japanese Circulation Society recommends a volumetric method which uses mitral annular diameters from apical four-chamber (A4C) and two-chamber (A2C) views. However, given the elliptical shape of the mitral annulus, use of apical long-axis (A3C) and A2C views, which reflect the anatomical long and short axes, may improve measurement accuracy. This study aimed to determine the optimal echocardiographic view combination for precise EROA quantification in degenerative MR (DMR). **Methods:** We retrospectively analyzed 98 patients with DMR who underwent both transthoracic echocardiography (TTE) and three-dimensional transesophageal echocardiography (3D TEE) within three months between April 2018 and December 2023. EROA was calculated using volumetric methods based on two TTE view combinations, A4C-A2C (EROA 4/2) and A3C-A2C (EROA 3/2). These were compared with 3D TEE-derived vena contracta area (VCA), which served as reference standard. **Results:** Mean values of EROA were 0.57 ± 0.23 cm^2^ for EROA 4/2, 0.50 ± 0.21 cm^2^ for EROA 3/2, and 0.49 ± 0.18 cm^2^ for 3D TEE VCA. EROA 4/2 was significantly larger than VCA (*p* < 0.01), whereas EROA 3/2 did not significantly differ from VCA (*p* = 0.41) and showed a stronger correlation with VCA than EROA 4/2 (r = 0.829 vs. r = 0.638, *p* < 0.01). **Conclusions:** Volumetric EROA assessment using A3C and A2C views provides more accurate quantification in DMR than the conventional A4C and A2C approach. Anatomically appropriate imaging planes should be prioritized to enhance the accuracy of MR severity evaluation.

## 1. Introduction

Degenerative mitral regurgitation (DMR) is a progressive valvular disorder which requires accurate quantitative assessment to guide therapeutic decisions and prevent progression to heart failure and arrhythmias. Effective regurgitant orifice area (EROA) is strongly correlated with clinical outcomes and guideline-based management thresholds and is accordingly considered the critical parameter for severity assessment [1]. The volumetric method, which calculates regurgitant volume as the difference between left ventricular and aortic forward stroke volumes, has theoretical advantages over the proximal isovelocity surface area (PISA) method due to its involvement of fewer geometric assumptions. However, current Japanese Circulation Society guidelines recommend calculating mitral valve annulus area (MVA) using diameters from apical four-chamber (A4C) and two-chamber (A2C) views, despite the mitral annulus being anatomically elliptical rather than circular [2]. This geometric mismatch between the imaging planes used and true anatomical axes may compromise measurement accuracy. Thus, the optimal combination of echocardiographic views for accurate volumetric EROA quantification in DMR remains unclear.

The anatomical complexity of the mitral annulus presents a fundamental challenge to accurate echocardiographic assessment. The mitral annulus forms an elliptical, saddle-shaped structure with distinct long and short axes that anatomically correspond to the apical long-axis (A3C) and two-chamber (A2C) views, respectively [3]. The conventional A4C view, while easily obtainable, captures an oblique plane that may not represent either true anatomical axis, which may introduce systematic measurement error. Moreover, accurate EROA calculation using the volumetric method depends on precise MVA estimation, as errors in annular measurement are propagated through the calculation formula: [(A4C diameter/2 × A2C diameter/2 × π × VTI MV) − (LVOT diameter/2)^2^ × π × VTI LVOT] ÷ VTI MR [2]. While three-dimensional transesophageal echocardiography (3D TEE) can directly measure MVA and vena contracta area (VCA) with high accuracy, its routine use is limited by invasiveness and resource constraints. Therefore, optimizing the transthoracic echocardiographic approach by selecting anatomically appropriate imaging planes could substantially improve the accuracy of volumetric EROA assessment in clinical practice.

Here, we investigated the optimal combination of echocardiographic imaging planes for accurate quantification of MVA and EROA in patients with DMR. We compared MVA measurements obtained using the conventional A4C-A2C view combination versus the anatomically aligned A3C-A2C combination on transthoracic echocardiography, using 3D TEE-derived MVA as the reference standard. Additionally, we validated EROA values calculated from each TTE-based approach against VCA obtained via 3D TEE, which served as the gold standard for assessing MR severity in 98 patients with moderate to severe DMR.

## 2. Materials and Methods

We retrospectively enrolled 112 patients diagnosed with moderate or greater DMR who underwent both TTE and 3D TEE within a three-month period between April 2018 and December 2023.

Patients were excluded if they had moderate or greater concomitant aortic regurgitation (*n* = 6), inadequate image quality or incomplete image sets (*n* = 7), or extensive mitral annular calcification (*n* = 1).

After applying these exclusion criteria, a total of 98 patients were included in the final analysis.

### 2.1. Transthoracic Echocardiography

TTE was performed as routine clinical practice using the EPIQ system and X5-1 transducers (Philips Medical Systems, Andover, MA, USA) or Vivid E9 and M5S transducers (GE Vingmed, Horten, Norway) by standard methods in accordance with the guidelines of the American Society of Echocardiography and European Association of Cardiovascular Imaging [4]. The mitral annulus diameter was measured from inner edge to inner edge using the hinge point between the annulus and the attachment of the valve leaflet tips on magnified mitral valve images. For the aortomitral curtain portion, the hinge point of the anterior leaflet was used as the annulus. Measurements were taken from early to mid-diastole when the valve was maximally open on AC4, AC2, and AC3 images, respectively.

### 2.2. Transesophageal Echocardiography

**Figure 1:**
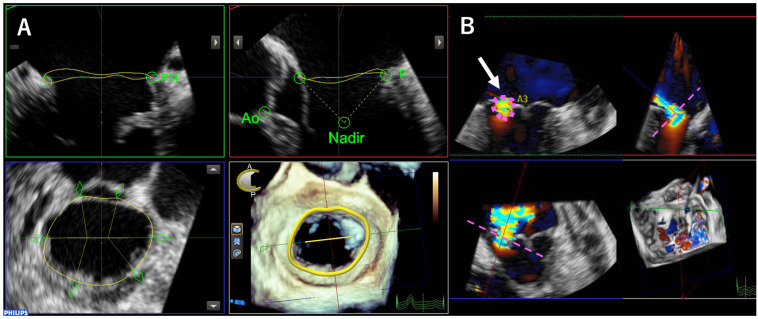
Methods of three-dimensional analysis of mitral valve using transesophageal echocardiography. From three-dimensional transesophageal echocardiographic (3D TEE) data, the mitral annulus area was measured using MVN software at mid-diastolic phase (**A**). Using the color 3D TEE data, the vena contracta area (VCA, white arrow) was evaluated using multiplanar reconstruction of the 3D TEE dataset. A cross-sectional plane perpendicular to the direction of the regurgitant jet was aligned through the vena contracta (pink dotted lines), and the VCA was measured by manual planimetry of the color Doppler flow signal obtained from an enface view (pink dotted circle) (**B**).

Three-dimensional TEE was performed using the EPIQ system with an X7-2t or X8-2t transducer (Philips Medical Systems, Andover, MA, USA). We obtained 3D full-volume MV data during breath-holding for six-beat acquisitions, or four heartbeats in patients with atrial fibrillation. The view was optimized for depth and gain setting before 3D acquisition for high spatial and temporal resolution data. For preoperative 3D data, we performed annulus analysis using MVN software, version 13 (Philips Medical Systems, Andover, MA, USA) (Figure 1A). VCA was evaluated using multiplanar reconstruction of the 3D TEE dataset. A cross-sectional plane perpendicular to the direction of the regurgitant jet was aligned through the vena contracta, and the VCA was measured by manual planimetry of the color Doppler flow signal obtained from an enface view (Figure 1B). To validate the accuracy of the VCA measurements, EROA was also calculated using the PISA method in 82 patients, excluding those with suboptimal PISA images, and the results were compared with the VCA values.

### 2.3. Statistical Analysis

Continuous variables are expressed as mean ± standard deviation and categorical variables are summarized as percentages and counts. Comparisons between two continuous variables, such as the MVA calculated by the TTE and that obtained by 3D TEE, as well as between EROA and VCA, were performed using Pearson correlation analysis. The comparison of correlation coefficients was performed using Steiger’s Z test. All statistical tests were two-sided, and a *p*-value of <0.05 was considered statistically significant. Agreement between EROA and VCA measurements was further assessed using Bland–Altman analysis. Fixed bias was determined based on whether the 95% confidence interval (95% CI) of the mean difference included zero. Inter- and intra-observer variability for annular diameter measurements on two-dimensional (2D) TTE were evaluated by having two independent observers measure annular dimensions in 20 randomly selected cases. Similarly, inter- and intraobserver variability for 3D TEE-derived MVA and VCA measurements were assessed in another set of 20 randomly selected cases, also measured by two other independent observers. These results were analyzed using intraclass correlation coefficients (ICC). All statistical analyses were performed using JMP Pro version 18 (SAS Institute Inc., Cary, NC, USA). This study was approved by Chiba University Hospital Ethics Committee (Reference no. HK202403-19).

## 3. Results

A total of 98 patients were included in the analysis. Mean age was 65 ± 13 years, and 56 patients (57%) were male. Among the 98 patients, 95 had severe DMR while 3 had moderate DMR. Patient characteristics and the average values of standard TTE parameters are summarized in Table 1.

### 3.1. Comparison of MVA Values by Measurement Method

**Figure 2:**
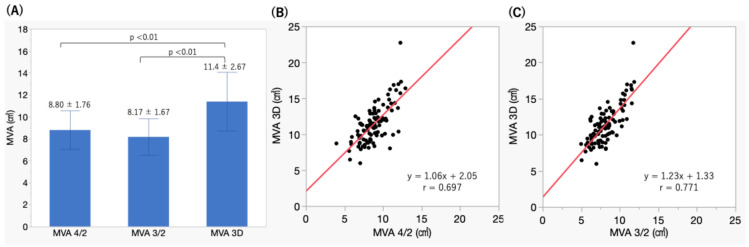
(**A**) Comparison of mitral valve annulus area values by measurement method. (**B**) The correlation between MVA 3D and MVA 4/2. (**C**) The correlation between MVA 3D and MVA 3/2. Red line; regression line.

The mean MVA values were as follows: 8.80 ± 1.76 cm^2^ for MVA calculated from the apical 4-chamber and 2-chamber views (MVA4/2), 8.17 ± 1.67 cm^2^ for MVA from the apical long-axis and 2-chamber views (MVA 3/2), and 11.4 ± 2.67 cm^2^ for MVA measured directly by 3D TEE. Both MVA 4/2 and MVA 3/2 were significantly smaller than the MVA obtained by 3D TEE (both *p* < 0.01) but demonstrated good correlation with the 3D measurements. The MVA 3/2 showed a significantly stronger correlation with VCA than EROA4/2. (r = 0.771 vs. r = 0.697, *p* = 0.028) (Figure 2A–C).

Mitral valve annulus area (MVA) measured directly by 3D TEE (3D MVA) was significantly larger than both the MVA calculated from the apical 4-chamber and 2-chamber views (MVA4/2) and the MVA from the apical long-axis and 2-chamber views (MVA 3/2) (A). Both the MVA 4/2 and MVA 3/2 demonstrated good correlation with the 3D MVA (B, C).

### 3.2. Comparison of EROA Measured by Different Methods with VCA

**Figure 3:**
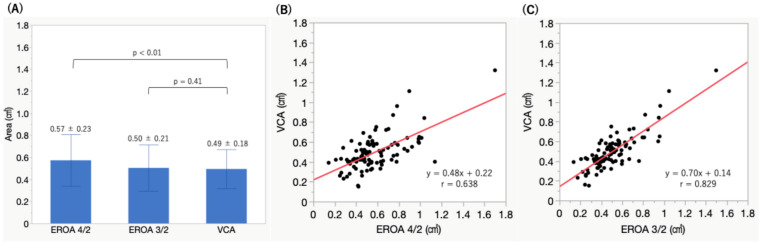
Comparison of effective regurgitant orifice area values by measurement method. Effective regurgitant orifice area using the A4C and A2C views (EROA 4/2) was significantly larger than the vena contracta area (VCA) measured by three-dimensional transesophageal echocardiography (**A**). EROA using the A3C and A2C views (EROA3/2) showed no significant difference compared to VCA and exhibited a stronger correlation with VCA (**B**) than the correlation between EROA 4/2 and VCA (**C**). Red line; regression line.

**Figure 4:**
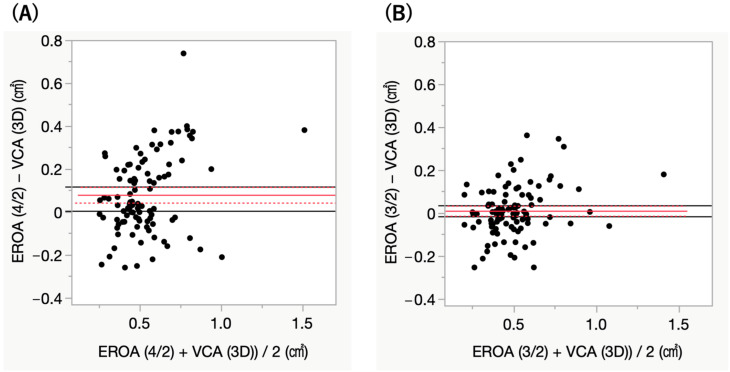
(**A**) Bland–Altman plot comparing EROA4/2 and VCA; (**B**) Bland–Altman plot comparing EROA3/2 and VCA. Effective regurgitant orifice area using the A3C and A2C views (EROA 3/2) demonstrated narrower limits of agreement with vena contracta area (VCA) than EROA using the A4C and A2C views (EROA 4/2) on Bland–Altman analysis. Red line; mean difference, red dash line; 95% confidence interval for the mean, black line; zero reference line.

**Figure 5:**
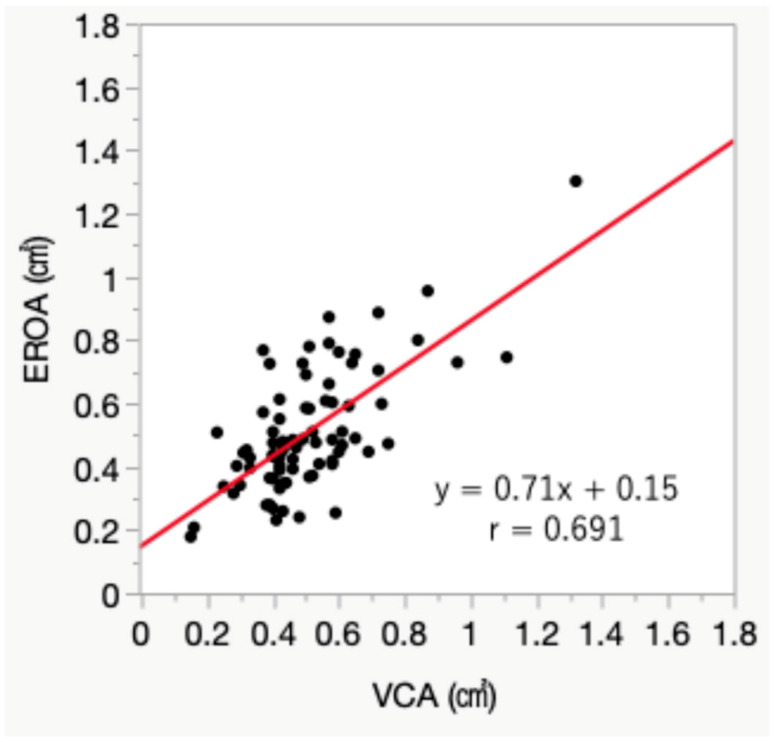
Correlation between vena contracta area and effective regurgitant orifice area measured by PISA method. Effective regurgitant orifice area (EROA) measured by the PISA method showed good correlation with vena contracta area measured using three-dimensional transesophageal echocardiography. Red line; regression line.

The mean values of EROA were 0.57 ± 0.23 cm^2^ for EROA calculated using the A4C and A2C views (EROA 4/2), 0.50 ± 0.21 cm^2^ for the A3C and A2C views (EROA 3/2), and 0.49 ± 0.18 cm^2^ for 3D TEE VCA (Figure 3A). EROA 4/2 was significantly larger than VCA (*p* < 0.01), demonstrating a fixed bias (95% CI: 0.042 to 0.12). In contrast, EROA 3/2 showed no significant difference compared to VCA (*p* = 0.41) and showed a stronger correlation with VCA than EROA 4/2 (r = 0.829 vs. r = 0.638, *p* < 0.01) (Figure 3B,C). Furthermore, EROA 3/2 demonstrated narrower limits of agreement with VCA than EROA 4/2 on Bland–Altman analysis (Figure 4A,B). Additionally, EROA measured by the PISA method showed good correlation with VCA (r = 0.691, *p* < 0.01) (Figure 5).

### 3.3. Inter- and Intraobserver Variability of Each Measurement

High inter- and intra-observer reliability was confirmed for annular diameter measurements across all TTE views, as well as for MVA and VCA measurements obtained by 3D TEE (Table 2).

## 4. Discussion

In this study, we found that volumetric EROA assessment using apical long-axis (A3C) and two-chamber (A2C) views provides significantly more accurate quantification of degenerative mitral regurgitation than the conventional four-chamber (A4C) and two-chamber approach, with EROA3/2 (0.50 ± 0.21 cm^2^) showing no significant difference from the reference standard 3D TEE vena contracta area and superior correlation. We also found that the conventional A4C-A2C method systematically overestimates mitral regurgitation severity. Furthermore, both measurement approaches showed high inter- and intra-observer reliability across all TTE views, with MVA3/2 demonstrating better correlation with 3D TEE-derived MVA than MVA4/2.

The vena contracta represents the narrowest portion of a regurgitant jet, occurring at or immediately downstream of the regurgitant orifice. The VCA, measured via 3D echocardiography, has been shown to correlate strongly with the EROA, a key metric of MR severity [5]. Furthermore, current guidelines recognize VCA as a reliable quantitative parameter because it remains relatively independent of flow rate and driving pressure [6]. While cardiac magnetic resonance imaging offers alternative quantitative methods for assessing MR [7,8], its routine clinical application is limited by high costs and time constraints. Similarly, although the PISA method is widely used in echocardiography, it is prone to hemodynamic influences and measurement errors depending on lesion morphology and location. VCA does have recognized limitations, particularly in the assessment of multiple regurgitant jets or non-holosystolic regurgitation. However, as these specific conditions were not present in the current study population, the reliability of VCA was considered well-maintained. Consequently, this study adopted VCA as the primary indicator of MR severity.

These findings have important clinical implications, given that accurate EROA quantification is critical for guideline-based management, namely that thresholds of ≥0.40 cm^2^ determine surgical intervention timing in severe DMR.

### 4.1. Clinical Importance of Quantitative Assessment in Mitral Regurgitation

EROA, a key parameter for evaluating the severity of MR, is known to be an independent predictor of both cardiac and all-cause mortality. Previous studies have reported that an EROA ≥ 0.40 cm^2^ is associated with a fivefold increase in mortality and a sixfold increase in the risk of cardiac events compared to patients with mild MR [9]. Therefore, accurate quantification of EROA is of great clinical importance.

In cases of DMR, EROA is typically assessed using the PISA method. However, in patients with multiple regurgitant jets or broad prolapsing segments, the PISA method tends to underestimate MR severity, limiting its applicability. In contrast, the volumetric method cannot be applied in the presence of significant aortic regurgitation but is otherwise broadly applicable in DMR regardless of prolapse location or extent [10]. Improving the accuracy of the volumetric method is thus a clinically relevant goal in the assessment of MR severity.

### 4.2. Clinical Utility of MVA and EROA Derived from Apical Long-Axis and Two-Chamber Views

Accurate evaluation of MVA is crucial not only for calculation of EROA but also understanding mitral valve function, planning surgical or transcatheter interventions, and determining appropriate device sizing. While advanced imaging modalities such as 3D TEE, cardiac computed tomography, and magnetic resonance imaging provide comprehensive spatial assessment of the mitral annulus [11], their routine use is limited by invasiveness, higher cost, and availability. TTE, with its widespread accessibility and real-time imaging capability, remains the most commonly used modality in clinical practice. Nonetheless, estimating MVA by 2D TTE has inherent limitations, including geometric assumptions, suboptimal image plane alignment, and significant operator dependency. These limitations often result in systematic underestimation compared to three-dimensional imaging modalities [12]. A major source of this error is attributable to the anatomical complexity of the mitral annulus, which is noncircular and saddle-shaped. Current guidelines recommend using diameters obtained from A4C and A2C views, assuming a circular or elliptical geometry. However, this simplification neglects commissure-to-commissure (CC) dimensions and localized asymmetry and thereby contributes to inaccurate area estimations [3]. A key step toward improving accuracy is ensuring alignment with anatomically correct imaging planes. The previous report demonstrated that when the 2D TTE image plane using CC diameter and antero-to-posterior (AP) diameter were aligned with the true annular axes, strong correlations with CT-derived measurements were observed (AP diameter: r = 0.96; CC diameter: r = 0.91) [13].

Given that the CC dimension closely corresponds to the diameter visualized in the AC2 view and that the AP dimension closely corresponds to the diameter visualized in the AC3 view, our results suggest that annular measurements derived from the A3C and A2C views yielded the best correlation with 3D reference values.

In the present study, although both combinations demonstrated a good correlation with the directly measured MVA, the absolute values of MVA derived from the A3C-A2C combination were significantly smaller than those measured by 3D TEE. This discrepancy is likely attributable to the use of transthoracic imaging, in which endocardial borders may appear unclear due to attenuation through the chest wall. As a result, annular measurements may be taken from points slightly within the true annular boundaries, leading to underestimation. Therefore, optimization of image quality is essential, including appropriate gain settings, focus adjustment, and the use of harmonic imaging to clearly delineate the mitral annular margins [14,15].

In addition, as illustrated in the previous report [16], the A4C view has a wider acquisition window, whereas the apical long-axis view requires alignment with the aortic valve and is therefore obtained within a narrower imaging plane. This narrower acquisition range reduces variability and may explain the lower intra- and interobserver variability observed with the A3C view (Table 2). These findings suggest that the A3C-A2C view combination is not only more accurate but also more suitable for routine clinical use in the volumetric assessment of DMR.

### 4.3. Considerations for Achieving More Accurate Quantitative Assessment

To further improve the accuracy of quantitative assessment, future investigations should also address the morphological characteristics of the left ventricular outflow tract (LVOT). Currently, the LVOT area used in EROA calculation is derived based on the assumption of a circular cross-sectional shape. However, the anatomical LVOT is typically elliptical, and the diameter measured in the parasternal long-axis view often corresponds more closely to the minor axis of the ellipse. As a result, the current method may lead to underestimation of the true LVOT area [16]. If TTE can be optimized to more accurately estimate the actual LVOT area, the precision of volumetric assessment, including EROA calculation, could be further improved.

### 4.4. Integration of Anatomical Accuracy and Modern Structural Heart Disease Treatment Planning

The findings of this study underscore that anatomically accurate morphological evaluation is not merely an academic exercise, but a clinical necessity that directly informs a workflow-oriented imaging strategy for modern transcatheter interventions. As transcatheter mitral valve implantation and edge-to-edge repair (TEER) continue to evolve, the “upstream” uncertainties—such as suboptimal device sizing or unpredictable hemodynamic outcomes—remain significant challenges [17]. Our approach, focusing on anatomically aligned annular diameters and derived EROA, provides a pragmatic solution to mitigate these uncertainties at the foundational level of procedural planning.

Recent literature supports this shift toward standardized, image-driven protocols. For instance, the implementation of standardized 3D-TEE maneuvers has been shown to enhance the robustness of measurements during TEER, ensuring consistency across different operators and time points [18]. This alignment between our methodology and such standardized approaches emphasizes that robust quantification is predicated on the identification of anatomically correct planes. By minimizing measurement variability through these standardized protocols, we facilitate a more reliable transition from diagnostic imaging to clinical decision-making.

Furthermore, the clinical trajectory is increasingly moving toward integrated, image-driven structural planning. The recent application of holographic mixed reality for transcatheter aortic valve replacement planning exemplifies how advanced visualization tools are being used to refine pre-procedural simulations [19]. However, the efficacy of such high-end “back-end” technologies is entirely dependent on the precision of the “front-end” quantitative data provided by echocardiography.

By ensuring the accuracy of these initial measurements, our study contributes to a more robust workflow that is applicable not only to aortic but also to complex mitral interventions. In conclusion, the integration of anatomically precise imaging into a standardized workflow is essential for reducing procedural risks and optimizing outcomes in the rapidly advancing field of structural heart disease.

### 4.5. Limitations

There are several limitations in this study. First, the study was conducted under a retrospective design with a relatively small number of patients, and the study may be underpowered for some analyses. Second, although A2C views were available in all patients, CC views were only acquired in a subset of cases. As a result, mitral annular diameters in this study were measured using A2C views. However, as discussed above, CC views would be more anatomically appropriate for this purpose. Future studies should aim to obtain CC views in all patients to allow for more accurate and comprehensive assessment. Finally, it should be acknowledged that the majority of patients in this study had severe MR, which may limit the generalizability of our findings to all grades of severity. In this study, MR severity was determined by board-certified members of the Japanese Society of Echocardiography through a comprehensive assessment. This included TTE parameters—such as the PISA method, volumetric methods, left atrial and left ventricular dimensions, and peak E-wave velocity—alongside VCA measured via TEE. Since the study population exclusively comprised patients undergoing both TTE and TEE for invasive treatment eligibility assessment, cases with less than moderate regurgitation were not evaluated. However, anatomically accurate measurement of the mitral annular diameter remains clinically valuable regardless of regurgitation severity. Furthermore, given the improving accuracy of 3D-TTE [20], future studies could provide clinically useful insights by examining the correlation between VCA and volumetric methods (A3C + A2C or A4C + A2C) using 3D-TTE, particularly in mild cases where TEE is not clinically indicated.

## 5. Conclusions

The combination of A3C and A2C views, especially in severe DMR, provides more accurate quantitative assessment of DMR using the volumetric method than the conventional A4C and A2C views.

## Figures and Tables

**Table 1:** Patient characteristics and echocardiographic parameters.

Characteristic	*n* = 98
Age (years)	65 ± 13
Males [n, (%)]	56 (57)
Body surface area (m^2^)	1.6 ± 0.2
MR severity	
Moderate	3 (3)
Severe	95 (97)
Hypertension	62 (63)
Dyslipidemia	62 (63)
Diabetes mellitus	28 (29)
Atrial fibrillation	21 (21)
Coronary artery disease	12 (12)
Transthoracic echocardiographic parameters	
LAD (mm)	47 ± 11
LVEDD (mm)	54 ± 6
LVESD (mm)	34 ± 6
LVEF (%)	64 ± 8

Data are expressed as mean ± SD or *n* (%). MR, mitral regurgitation; LAD, left atrial diameter; LVEDD, left ventricular end-diastolic diameter; LVESD, left ventricular end-systolic diameter; LVEF, left ventricular ejection fraction.

**Table 2:** Inter- and Intraobserver Variability of Measurements.

Transthoracic Echocardiography			
Annular Diameter	A4C View	A2C View	A3C View
ICC (1, 3)	0.94	0.97	0.97
ICC (2, 3)	0.85	0.82	0.84
Three-dimensional transesophageal echocardiography			
	MVA	VCA	
ICC (1, 3)	0.96	0.94	
ICC (2, 3)	0.92	0.89	

A4C, apical four-chamber; A2C, apical two-chamber; A3C, apical three-chamber; MVA, mitral valve annulus area; VCA, vena contracta area.

## Data Availability

No data will be shared related to this study.
